# Taurine as a Natural Antioxidant: From Direct Antioxidant Effects to Protective Action in Various Toxicological Models

**DOI:** 10.3390/antiox10121876

**Published:** 2021-11-24

**Authors:** Peter F. Surai, Katie Earle-Payne, Michael T. Kidd

**Affiliations:** 1 Vitagene and Health Research Centre, Bristol BS4 2RS, UK; 2 Department of Microbiology and Biochemistry, Faculty of Veterinary Medicine, Trakia University, 6000 Stara Zagora, Bulgaria; 3 Biochemistry and Physiology Department, Saint-Petersburg State University of Veterinary Medicine, 196084 St. Petersburg, Russia; 4 Department of Animal Nutrition, Faculty of Agricultural and Environmental Sciences, Szent Istvan University, H-2103 Gödöllo, Hungary; 5 NHS Greater Glasgow and Clyde, Renfrewshire Health and Social Care Centre, 10 Ferry Road, Renfrew PA4 8RU, UK; katie.earle-payne@ggc.scot.nhs.uk; 6 Center of Excellence for Poultry Science, University of Arkansas, Fayetteville, AR 72701, USA; mkidd@uark.edu

**Keywords:** antioxidant, free radicals, mitochondria, stress, taurine

## Abstract

Natural antioxidants have received tremendous attention over the last 3 decades. At the same time, the attitude to free radicals is slowly changing, and their signalling role in adaptation to stress has recently received a lot of attention. Among many different antioxidants in the body, taurine (Tau), a sulphur-containing non-proteinogenic β-amino acid, is shown to have a special place as an important natural modulator of the antioxidant defence networks. Indeed, Tau is synthesised in most mammals and birds, and the Tau requirement is met by both synthesis and food/feed supply. From the analysis of recent data, it could be concluded that the direct antioxidant effect of Tau due to scavenging free radicals is limited and could be expected only in a few mammalian/avian tissues (e.g., heart and eye) with comparatively high (>15–20 mM) Tau concentrations. The stabilising effects of Tau on mitochondria, a prime site of free radical formation, are characterised and deserve more attention. Tau deficiency has been shown to compromise the electron transport chain in mitochondria and significantly increase free radical production. It seems likely that by maintaining the optimal Tau status of mitochondria, it is possible to control free radical production. Tau’s antioxidant protective action is of great importance in various stress conditions in human life, and is related to commercial animal and poultry production. In various in vitro and in vivo toxicological models, Tau showed AO protective effects. The membrane-stabilizing effects, inhibiting effects on ROS-producing enzymes, as well as the indirect AO effects of Tau via redox balance maintenance associated with the modulation of various transcription factors (e.g., Nrf2 and NF-κB) and vitagenes could also contribute to its protective action in stress conditions, and thus deserve more attention.

## 1. Introduction

Among the many different nutrients essential for human/animal life, natural antioxidants have received tremendous attention over the last 3 decades. It is believed that activated oxygen (reactive oxygen species, ROS) and nitrogen (reactive nitrogen species, RNS) molecules are involved in fundamental processes of cell biology, including cell signalling and adaptation to stressors [1,2,3]. It is clear that well-controlled low levels of ROS and RNS are essential for homeostasis maintenance; however, increased and poorly controlled ROS and RNS production can lead to oxidative stress, an important cause of various human and animal diseases, decrease human quality of life, and decrease the productive and reproductive performance of farm animals [4,5]. During evolution in an oxygenated atmosphere, antioxidant defence systems, responsible for the prevention of damage by ROS and RNS and the adaptation to stresses, have been developed and shaped. Recent data on involvement of various transcription factors (e.g., Nrf2, NF-κB, etc.) and vitagenes in antioxidant defences changed attitudes towards nutritional antioxidants [1,2,3]. Traditional dietary antioxidants (vitamins E and C) and plant-derived natural antioxidants (carotenoids, polyphenols, etc.) are considered to be an important part of the external support of the antioxidant defence network. This defensive system is based mainly on antioxidant enzymes (SOD, GPx, GST, TR, Catalase, etc.) and low-molecular weight antioxidants (GSH, thioredoxin, ubiquinones, etc.) synthesised in human/animal tissues and often upregulated in response to moderate stress [1]. Deeper understanding of the interactions within the antioxidant defence network and approaches to redox balance maintenance [6,7] introduced new entrants into the antioxidant family, including carnitine [8,9,10] and taurine (Tau) [11,12,13], which are synthesised in the body, but also obtained from feed/food ingredients. In stress conditions when antioxidant requirement increases, above their dietary supply, assimilation and metabolism can be compromised; the roles of such antioxidants as Tau and carnitine deserve more attention [1]. In the recent aforementioned reviews, the antioxidant properties of Tau were described, but molecular mechanisms of its action remain elusive. Importantly, practically all antioxidant-related research on Tau was conducted with model systems based on mice and rats, and only recently, Tau received limited attention as an antioxidant and anti-inflammatory compound for poultry [14,15,16,17]. Therefore, there is a need for a more systematic approach to the antioxidant properties/action of Tau to cover its major direct and indirect involvements in the antioxidant network regulation, with specific emphasis on its possible application in human and animal nutrition. Recently, we reviewed in detail the antioxidant properties of carnitine [8,9,10] and silymarin [18]. The aim of this review is to systemise the antioxidant actions of taurine based on recent understandings of the antioxidant system’s operation and regulation in physiological and stress conditions by nutritional means. In particular, in this review, taurine metabolism, its direct antioxidant properties, mitochondria- and membrane-stabilizing properties, as well as protective effects in various toxicological models will be considered.

## 2. Taurine Sources

In 1827, a new compound was isolated from ox bile by German scientists Friedrich Tiedemann and Leopold Gmelin [19], which is now known as Tau (2-aminoethanesulfonic acid). It has a molar mass of 125.15 g/mol, and its molecular formula is C_2_H_7_NO_3_S. Tau is commonly called an amino acid, but it does not contain a carboxyl group and its amino group is located at the β-position. Furthermore, Tau is shown not to be incorporated into proteins, but extensively distributed in animal tissues [11]. Human and animals, including farm animals and poultry, obtain Tau from their diet and from endogenous synthesis. Food/feed ingredients of animal origin, including fish, dairy products and human milk, are the main natural dietary sources of Tau [20]. Interestingly, terrestrial plants including grains (major feed ingredients for farm animals/poultry) are practically Tau-free [21]. Tau synthesis in vivo is reported to be species-specific. In particular, rodents have a high synthetic capacity, while humans are characterised by an intermediate capacity to synthesise Tau, and cats lack the ability to synthesise this amino acid due to the limited activity of a key enzyme, namely cysteine sulfinate decarboxylase [22]. Furthermore, about 80% of the cysteine pool is known to be converted into Tau in rats, while only 20% is converted in cats [23]. It is demonstrated that Tau synthesis in animals occurs primarily in the liver from cysteine catalysed by cysteine dioxygenase and cysteine sulfinate decarboxylase. In addition, Tau synthesis has been discovered to occur in other tissues, including the brain, lungs, skeletal muscle and adipose tissue (for a review, see [24]).

It is believed that Tau biosynthesis cannot meet its requirement in mammals and birds, and dietary Tau intake is absolutely essential for Tau homeostasis [23]. Furthermore, there are some physiological states in which Tau synthesis is not adequate to meet physiological requirements. For example, newborn mammals are shown to be unable to synthesise sufficient amounts of Tau and therefore heavily rely on dietary Tau supply [24]. In mammals, depending on the Tau turnover rate, tissues can be divided into three groups. The first group includes the liver, kidney and pancreas, tissues with a fast Tau turnover rate (<1 day). The lung, spleen, intestine, testes, and bone marrow are characterised by a medium rate of Tau turnover (2–3 days), and comprise a second group. Furthermore, the third group combines tissues with the slowest rate of Tau turnover (3–7 days) and includes the brain, heart and skeletal muscles [25,26].

## 3. Taurine Essentiality and Requirement

Tau is known to be an essential nutrient for several animal species, including the cat, certain dogs, the fox, some monkeys and the anteater [27]. In major mammalian and avian species, Tau can be synthesised, but the yield is usually inadequate to meet its requirements, and thus dietary consumption of Tau is necessary to maintain its homeostasis [28]. Furthermore, in stress conditions, Tau synthesis could be inadequate or compromised, suggesting that Tau is an essential or semi-essential nutrient for many mammals and birds. The defects associated with Tau deficiency have been shown to affect major body tissues/systems and lead to retinal degeneration, cardiac dysfunction, immune deficiency, muscle atrophy, premature aging and impaired reproduction [27]. Interestingly, recent results demonstrate that Tau depletion could impair ammonia detoxification in mouse livers, associated with oxidative stress and senescence [29]. On the other hand, dietary supplementation of Tau was shown to be associated with a reduction in the risk of various diseases, including diabetes [30,31], metabolic syndrome [32], skeletal muscle disorders [33], retinal degeneration [23], atherosclerosis [34], liver diseases [35], central nervous system disorders [36], etc.

## 4. Taurine Absorption and Metabolism

Dietary Tau is well absorbed from the gastrointestinal tract, with bioavailability of about 90% [37]. After absorption, Tau is delivered to enterocytes via carrier mediated active transport in the brush border membrane. Next, Tau appears in the portal vein, is transported to the liver and released into the circulation. In general, the process of Tau absorption is believed to be quite fast. For example, in humans, plasma Tau concentrations reached a peak of 0.53 mM just 1.5 h after oral administration of Tau. Plasma elimination half-life of Tau is about 1.0 h [38]. It seems likely that in rats, Tau absorption is dose-dependent. For instance, the maximal plasma Tau concentrations were achieved at 15 and 45 min after a single oral low- or high-dose administration, respectively [39]. It is reported that Tau can enter cells via different mechanisms, including the high affinity and low transport capacity Na^+^-Cl^−^-Tau symport transporter or via the low affinity but high transport capacity proton-coupled amino acid transporter (PAT1; for a review, see [12]). Tau transporter (*TauT*) expression is dependent on the Tau status of the cells. In fact, a high Tau concentration is found to downregulate *TauT*, while low Tau status could upregulate *TauT*. It should also be mentioned that Tau could be reabsorbed into circulation through the renal tubules in the kidney. Several important features of *TauT* have been demonstrated [40,41]:

- the renal adaptive regulation of *TauT* depends on taurine availability and controls the body pool of Tau;
- renal Tau accumulation is affected by the ionic environment, electrochemical charge and pH;
- Tau transfer across the cell membrane is dependent on *TauT* and controlled by PKC phosphorylation post-translationally;
- p53 suppresses *TauT* expression;
- over-expression of *TauT* is shown to have protective effects on renal cells against oxidative stress-induced nephrotoxicity.
- high *TauT* expression was shown in the liver, kidney, brain, retinas and placenta of mammals [42].

Regulation of *TauT* by p53 and c-Jun was demonstrated by various methodologies including reporter gene assay, DNA binding, Western blot analysis, and immunohistochemistry [43]. Actually, *TauT* was detected to be down-regulated by p53 and up-regulated by c-Jun. Indeed, the inhibition of c-Jun N-terminal kinase (JNK) was associated with increased *TauT* promoter activity. Furthermore, overexpression of *TauT* is found to have protective effects against cisplatin-induced kidney damage in a *TauT* transgenic mouse model [43]. Interestingly, Tau uptake in the brush-border membrane of human intestinal cells takes place by means of both PAT1- and *TauT*-mediated transport. Under physiological conditions, *TauT*-mediated uptake is likely to be the major absorptive mechanism at low Tau concentrations. However, at higher Tau concentrations, PAT1-mediated uptake predominates [44].

Since Tau is not incorporated in proteins, the concentration of this nutrient is comparatively high in many animal tissues. Notably, its highest concentration has been detected in the retina (from 10 to 52 mM) and in the photoreceptor cell layer (80 mM; [45]), leucocytes (up to 50 mM; [46]), heart (10–40 mM), skeletal muscles (2–15 mM), and brain (2–6 mM; [47]). At the same time, the Tau concentration in the plasma and extracellular fluids is much lower, typically comprising 0.05–0.2 mM [48]. Usually, the highest Tau concentrations can be determined in highly energy-consuming tissues, including retina, nerves, kidney, heart, and oxidative muscle tissue [49]. Noteworthily, the levels of Tau in the heart are shown to be extremely high, ranging from 3 mM in cows to 40 mM in mice [50]. Tau was found to be excreted predominantly (95%) in urine as unmetabolised Tau (70%) and as a sulphate (about 25%), probably reflecting possible bacterial degradation of Tau in the intestine [51].

## 5. Biological Roles of Tau

Since the discovery of Tau 194 years ago, its various functions in skeletal muscle, the retina, the central nervous and cardiovascular systems have been shown [13]. The major biological functions of Tau are summarised in Table 1.

It is clear that Tau participates in the regulation of major biochemical pathways and physiological function in the body, being an important modulator of basic processes, including osmotic pressure, cation homeostasis, enzyme activity, receptor regulation, cell development and cell signalling [100]. In a recent review of the molecular mechanisms of the cytoprotective actions of Tau, regulation of the antioxidant defence network, energy metabolism, gene expression, ER stress, Ca^2+^-homeostasis as well as neuromodulation and osmoregulation were considered [13]. Recent transcriptome studies also provided evidence that Tau may play a role in the regulation of glycerophosphocholine metabolism, NO synthesis, fatty acid oxidation, ketone body degradation, and branched-chain amino acid metabolism in animal tissues, including the heart [101].

To investigate the biological role of Tau in animals, two major approaches are used. On the one hand, the consequences of Tau deficiency caused by its low dietary provision in species that are unable to synthesise this amino acid (cats) are very important in this regard. On the other hand, detrimental consequences due to the specific knockout of genes involved in Tau synthesis, transport and cellular Tau uptake are also of great importance. Recent studies into the Tau-deficient phenotypes indicate that they are characterised by impairments of growth, reproduction, energy metabolism and skeletal muscle function, cardiopathy and retinal degeneration [11]. Importantly, it has also been shown that the performance of transgenic mice lacking the taurine transporter (*TauT*KO) was dramatically compromised. For example, exercise endurance time was reduced more than 10 times, while the duration of running time to exhaustion was reduced more than 3-fold. Furthermore, the total running distance to exhaustion on the treadmill was shown to be reduced by more than 80% [102]. At the same time, whole-body *TauT* knockout mice were characterised by a significantly decreased Tau level in tissues with severe intolerance to exercise [103]. Furthermore, mitochondrial ROS generation was significantly increased in Tau-deficient hearts and ultrastructural abnormalities were found in the muscles of *TauT*KO mice [104,105]. In Tau-deficient (*TauT*KO) mice the levels of ubiquitinated protein were also significantly elevated [106]. Indeed, Tau deficiency/inadequacy is likely to increase animal susceptibility to various stresses. Therefore, it was hypothesised that the Tau-deficient phenotypes are primarily associated with the inadequate cytoprotective actions of Tau leading to changes in cell volume regulation, calcium homeostasis, membrane stabilisation and antioxidant defences [11]. In the next sections of this paper, evidence will be provided to substantiate the claim that improved regulation of the antioxidant defence network is the major mechanism responsible for the beneficial effects of dietary Tau supplementation.

## 6. Antioxidant Systems of the Body

During evolution in an oxygenated atmosphere, living organisms have successfully developed specific antioxidant protective mechanisms to deal with ROS and RNS [107]. This development was the major adaptive mechanism to enable animals to survive in an oxygen-rich environment. The general terms “antioxidant systems” or “antioxidant system network” combine these diverse mechanisms, responsible for the protection of cells from the damaging actions of ROS and RNS. They are also deeply involved in providing optimal conditions for cell signalling and adaptation to various stresses. The protective antioxidant compounds are shown to be located in major organelles, subcellular compartments, as well as in the extracellular space providing effective AO protection to occur. To be effective, the antioxidant defence network is based on several important mechanisms, including [1]:

- decreasing the localised oxygen concentration;
- reducing the activity of prooxidant enzymes;
- improving the efficiency of ETC in the mitochondria and decreasing electron leakage;
- inducing various transcription factors (e.g., Nrf2, NF-κB, etc.) with increased antioxidant response element-related synthesis of various antioxidants;
- binding metal ions (metal-binding proteins) and metal chelating;
- decomposing peroxides;
- chain breaking by scavenging intermediate radicals, including peroxyl and alkoxyl radicals;
- binding reactive products of peroxidation, such as MDA, 4-hydroxynoinenal, etc.
- repairing and removing damaged molecules;
- maintaining optimal redox status;
- maintaining redox signalling and vitagene activation with synthesis and increased expression of protective molecules;
- providing antioxidant recycling mechanisms, including vitamin E recycling;
- inducing protein glutathionylation as a way to prevent its irreversible oxidation;
- activating apoptosis/ferroptosis with the removal of terminally damaged cells and restricting mutagenesis.

It is important to underline that all antioxidants in the body are working as a ‘team’ providing effective antioxidant defence. There are important interactions within the team, when one member helps the other to work efficiently. It seems likely that a central role in antioxidant system regulation belongs to vitagene expression and the additional synthesis of protective molecules during stress conditions, leading to an improved ability to adapt to stress [1]. Certainly, ROS in limited amounts are considered essential secondary messengers, whereas in larger quantities, they cause detrimental changes to cell function associated with cell damage and cell death.

## 7. Antioxidant Properties of Tau

It is demonstrated that Tau contributes to the antioxidant defences in different ways, including [1,17,57]:

1. direct free radical scavenging;
2. maintenance of the integrity of electron-transport chain of mitochondria in stress conditions;
3. stabilizing biological membranes;
4. inhibiting ROS-generating specific enzymes;
5. modulating transcription factors responsible for upregulation and increased synthesis of AO enzymes;
6. up-regulating vitagenes (SOD, HSP, thioredoxin, TR and sirtuins), leading to improved adaptation to stress.

In the following sections, the first four mechanisms will be considered.

### 7.1. Direct Free Radical Scavenging

The antioxidant activities of Tau are of great interest in medical sciences [108]. However, Tau’s reactivity with superoxide, hydrogen peroxide, and hydroxyl radicals was shown to be quite poor [109,110]. Therefore, it seems likely that in physiologically relevant concentrations (1.5–3 mM), Tau cannot effectively scavenge such RONS as hydrogen peroxide, superoxide, or peroxynitrite [109,111,112]. Furthermore, IC50 values (the concentration inhibiting 50% of free radical generation) of Tau against hydroxyl radical, alkyl radical and DPPH were indicated to be above 4 mg/mL (>30 mM; [113]).

However, higher Tau concentrations (15–60 mM) showed free-radical scavenging activity in various in vitro systems. For instance, based on the results of the registration of changes in luminol luminescence caused by superoxide generated in the hypoxanthine/xanthine oxidase model system or by peroxide formation from the glucose/glucose oxidase system, it was proven that Tau (30 mM) possessed superoxide and H_2_O_2-_scavenging activity [114]. In the same study, spin trapping clearly demonstrated the superoxide scavenging activity of Tau. In a later study, Tau in concentrations >15 mM was reported to scavenge peroxyl radical and superoxide anion as well as nitric oxide and peroxynitrite [115], but Tau was not effective in a reaction with 1 mM H_2_O_2_ [52]. In a model system based on the thermal decomposition of AAPH (the peroxyl radical generator), Tau (60 mM) was found to completely block this process. Furthermore, the authors clearly demonstrated other AO activities of Tau associated with the amelioration of *t*-BHP-induced damage to lipids in liver slices and responsible for redox balance maintenance through the protection of -SH groups and the effective preservation of -SH pool in oxidative stress [115]. Tau was able to mitigate the peroxinitrite-induced formation of nitrotyrosine adducts and prevent the reduction in SOD activity [115]. Later, it was reported that Tau possessed a dose-dependent DPPH radical scavenging activity with an IC50 of about 1.2 mM. Interestingly, in the same study, it was shown that the IC50 values of alkyl, hydroxyl, and superoxide radical scavenging activities were 0.206 mg/mL (~1.7 mM), 0.241 mg/mL (~1.9 mM), and >8 mM, respectively [54]. Therefore, it could be concluded that free radical scavenging activity of Tau is condition-dependent. Furthermore, the superoxide radical scavenging activity by Tau is likely to be lower than that for other radicals, including DPPH, hydroxyl or alkyl radicals. Interestingly, it was demonstrated in vitro that Tau and hypotaurine can react with superoxide anions to form the novel molecule called peroxytaurine [52]. Importantly, Tau can be considered as a DNA protector preventing DNA damage under various stress conditions [54,116]. In general, in plasma and most tissues of humans and farm animals/poultry, Tau concentrations > 15 mM are most likely not achievable. However, in such tissues as heart and eye lenses characterised by high Tau concentrations, the direct free-radical scavenging activity of Tau is likely to be important. The aforementioned data demonstrate that in biological systems, the direct ROS scavenging activity of Tau is quite weak, and other regulatory activities of Tau in the antioxidant defence network are probably of great value, and they will be considered in the following sections of this paper.

It seems likely that Tau could play a protective role, including free-radical scavenging, in various phagocyte cells, including neutrophils. Tau can react with hypochlorous acid, forming taurine chloramine (TauCl, [109]), which is less toxic than hypochlorous acid and can suppress the production of several pro-inflammatory mediators via the downregulation of NF-κB [92], leading to effective modulation of the immune system. In activated macrophages in vitro, Tau and TauCl were reported to inhibit the generation/expression of nitric oxide, prostaglandin E, tumour necrosis factor, and interleukin, regulating the inflammatory response and preventing damage to immune cells [117]. Therefore, in inflammatory cells/tissues, activated neutrophils after apoptosis could release TauCl, leading to suppression of the production/expression of pro-inflammatory mediators including superoxide anion, NO, interleukins, TNF-α and prostaglandins. TauCl released from activated neutrophils could also help in cell recovery from inflammation-associated oxidative stresses. At the same time in macrophages, TauCl could be responsible for enhanced expressions of various antioxidants, including HO-1, peroxiredoxin, thioredoxin, GPx, and CAT [93].

### 7.2. Protective Effects of Tau on Mitochondria

It is generally accepted that mitochondria are the primary cellular consumers of oxygen and the main free radical source in the cell, and ROS-producing mitochondrial enzymes include [118]:

- the electron-transport chain (ETC), including complexes I, II and III;
- pyruvate dehydrogenase and glycerol-3-phosphate dehydrogenase;
- α-ketoglutarate dehydrogenase and aconitase;
- cytochrome b5 reductase;
- dihydroorotate dehydrogenase;
- the monoamine oxidases A and B.

Complex I (ubiquinone reduction site), complex III (the outer quinone-binding site of the Q-cycle) and glycerol 3-phosphate dehydrogenase are reported to be major sources of ROS production [119], and there are at least eleven sites of ROS production in mammalian mitochondria [120,121]. Furthermore, various environmental and nutritional stresses imposing oxidative stress can cause mitochondrial dysfunction with substantially enhanced ROS production, leading to increased lipid peroxidation, protein oxidation as well as mitochondrial DNA damage [122]. The detrimental effects of ROS have been a focus of research for many years, but recently it has become obvious that mitochondrially generated ROS actively participate in the regulation of intracellular signal transduction pathways [123,124], helping cell adaptation to stress [125]. Tau is found in the mitochondria [105,126,127,128] in comparatively high (20–30 mM) concentrations [59], and these findings represent important steps for the elucidation of the crucial regulatory roles of Tau in mitochondrial functions.

To study the molecular mechanisms responsible for the antioxidant activity of Tau, specific Tau antagonists and Tau transport inhibitors, including β-alanine, were widely used [58]. The authors showed that adding β-alanine to the medium used for cultivation of isolated cardiomyocytes caused a significant (by 45%) reduction in Tau associated with a substantial increase in mitochondrial oxidative stress. In the aforementioned experiment, the activities of ETC complexes I and III were reported to be decreased (by 50–65%). Furthermore, impaired synthesis of key subunits of the ETC (e.g., ND5 and ND6) and enhanced mitochondrial ROS production were observed due to Tau deficiency. Co-administration of Tau with β-alanine resulted in the restoration of Tau concentrations in mitochondria, leading to the amelioration of oxidative stress-related changes [58]. The authors concluded that by improving the mitochondrial synthesis of proteins participating in the assembly of active respiratory chain complexes and maintaining ETC activity, Tau can help the mitochondria, controlling ROS generation and ameliorating oxidative stress.

Tau has been reported to participate in the regulation of the correct translation and expression of the proteins of the mitochondrial respiratory chain. Importantly, in mitochondrial transfer RNA (tRNA), Tau with modified uridine has been discovered [129]. Therefore, taurine-modified tRNA has been suggested to play an essential role in the stabilisation of codon-anticodon interactions, an important event in the translation of proteins of ETC [130]. A mitochondrial protein MTO1 that shares evolutionary conservation with proteins, such as yeast mto1, has been demonstrated to participate in protein synthesis regulation via tRNA modification. Indeed, the deficiency of MTO1 caused embryonic lethality in mice at a very early developmental stage as a result of a loss of Tau modification of mt-tRNA [131]. Data demonstrating that MTO1 deficiency leads to compromised mitochondrial translation and respiratory activity confirmed the crucial role of Tau modification in protein homeostasis and mitochondrial translation regulation in the cell. Indeed, mitochondria with abnormal morphology, aggregated and misfolded proteins were observed in MTO1-deficient cells [131]. Therefore, under stress conditions, Tau depletion in various tissues could result in a reduction in Tau-modified tRNA with subsequent damage to ETC, causing the induction of ROS production and further mitochondrial dysfunction. It could be concluded that by regulating mitochondria integrity and functionality, Tau could improve AO defences and suppress the ROS production in the cell [132]. On the other hand, in Tau-deficient tissues, mitochondrial ROS production could be induced [104,105,106] due to the defective translation of mitochondrial encoded proteins and ETC impairment [27,132]. Mitochondrial ROS generation was demonstrated to be enhanced in Tau-deficient hearts [104,105,106], and Tau transporter knockout (taut−/−) mice were reported to show hepatic mitochondria dysregulation with severely decreased respiratory control ratio [133]. In cardiomyocytes from transgenic mice with knockout for the Tau transporter, suppressed ATP production and increased superoxide generation were observed [104]. In general, Tau dietary supply/therapy is an important approach to deal with oxidative stress by regulating mitochondrial protein biosynthesis and restoring mitochondrial function [27,134].

Furthermore, by increasing GSH concentrations in the mitochondria, Tau can effectively regulate/maintain an optimal redox balance in this organelle [135,136]. It is widely accepted that maintaining the antioxidant defence network’s integrity and preserving physiological redox balance in major organs/tissues of human and animals are important elements of health maintenance [137]. Tau can be considered as an important modulator of the mitochondrial matrix buffer responsible for the mitochondrial pH gradient and mitochondrial homeostasis stabilisation/maintenance [48] and regulating the activity of specific enzymes including pyruvate dehydrogenase [136]. Tau could also regulate mitochondrial calcium homeostasis, stimulate mitochondrial Mn-SOD, and preserve mitochondrial function [133,138]. The amelioration of cytoplasmic calcium increasing as a result of the prevention of mitochondrial dysfunction by Tau could be an additional molecular mechanism of AO action of this nutrient [139]. Therefore, Tau protects mitochondrial integrity and homeostasis as a result of the amelioration of intracellular calcium overload [139,140]. Importantly, in a range of in vitro studies with cardiomyocytes cultured in Tau-deficient, medium damage to ETC leading to excessive ROS production was responsible for oxidative stress [141,142]. At the same time, adding Tau into the medium made it possible to restore the ETC’s integrity and to suppress ROS production [58]. Furthermore, Tau is reported to reduce oxidative stress in various model systems by ameliorating damage to ROS-sensitive AO enzymes [13].

In stress conditions, mitochondrial stabilisation by Tau maintains mitochondrial homeostasis and redox balance, leading to a reduction in the leakage of ROS produced inside mitochondria [48]. For instance, in Mn-exposed mitochondria, the deterioration of the brain mitochondrial membrane potential was shown to result in the reduction in mitochondrial dehydrogenases activity, increasing mitochondrial swelling, and depleting mitochondrial ATP. In such conditions, Tau added to the medium at physiological concentrations (0.1–10 mM) was reported to ameliorate the aforementioned detrimental changes [143]. In a traumatic brain injury model, Tau was also demonstrated to improve antioxidant defence mechanisms as a result of increasing mitochondrial ETC activity [144]. In particular, in an in vitro model based on mouse muscle cells, the pre-treatment with 0.1 µM Tau was found to reduce MDA-induced cell death rate as a result of mitochondrial membrane potential restoration, improved ATP production, decreased protein adduct formation and enhanced AO defences, as evidenced by increased expression of Nrf2 and the factors of mitochondrial biogenesis [145]. The authors suggested that an induction of mitochondrial biogenesis could be responsible for the cytoprotective effects of Tau. Importantly, oxidised proteins and amino acids can cause oxidative stress [146]. Furthermore, in rabbits and cultured osteocytes, Tau was shown to inhibit glucocorticoid-induced bone mitochondrial injury and to mitigate osteonecrosis [147]. Pre-treatment of mouse testicular mitochondria, exposed to bisphenol A, with 30 and 50 µmol/L of Tau was reported to suppress mitochondrial oxidative stress and to restore mitochondrial membrane potential [148].

Further evidence on the protective effects of Tau in maintaining mitochondria integrity and homeostasis, including redox balance, came from studies with various chemicals that are strong promoters of the oxidative stress in the mitochondria, which will be considered in the following sections of this paper. Tau treatment was demonstrated to reduce/ameliorate the detrimental consequences of toxicologic oxidative stress [13,61,132].

The antioxidant properties of Tau have been widely investigated in various model systems. It was concluded that Tau’s protective actions could be related to the maintenance/preservation of mitochondrial integrity and functions in various stress conditions. Furthermore, Tau supplementation is proven to be effective in protecting against human pathologies associated with oxidative stress due to mitochondrial defects including various mitochondrial diseases, cancer, neurological disorders, cardiovascular diseases, metabolic syndrome, and aging [60].

### 7.3. Membrane-Stabilizing Activity of Tau

In 1973, Huxtable and Bressler [149] suggested that taurine could stabilise biological membranes. Later, in an in vitro study that employed isolated segments of frog retinas, the membrane-stabilizing properties of Tau were demonstrated [150]. This effect was also confirmed in retinoid-treated lymphoblastoid cells in culture [151]. Indeed, photoreceptor membranes were shown to be stabilised by Tau, and this nutrient could be considered as a physiological membrane stabiliser [152]. In fact, Tau can bind to the membrane at or near the insulin receptor location, with subsequent hypoglycaemic effects in rats [153]. The inhibition of LP in biological membranes could result in their stabilisation. For example, Tau (25 mM) was reported to alleviate the increase in LP in the light-induced eye damage model [154]. Tau was also demonstrated to inhibit protein phosphorylation in rat retinal membranes [155]. Leibowitz et al. [156] showed that Tau was able to suppress phosphate incorporation into rat retinal proteins. It has been proposed that increasing the PE/PC ratio due to Tau supplementation could lead to the modulation of the cell membrane’s fluidity, affecting membrane stability and integrity [157]. Possible mechanisms of the stabilizing effects of Tau on biological membranes were outlined by Schaffer et al. [56] and can be summarised as follows. Firstly, the inhibition of lipid peroxidation and reduction in oxidative injury to membranes could be responsible for the cytoprotective effects of Tau. The regulation of protein phosphorylation by Tau could change membrane structure and affect protein activities and membrane functions, including Ca transport. Membrane structure and functions are also dependent on Tau interactions with neutral phospholipids and inhibition of phospholipid N-methylation by Tau [56]. Tau-dependent membrane stabilisation and maintenance of permeability to water and ions under various stress conditions could be responsible for the protection against various toxicants, including streptozotocin [46,158].

As mentioned above, uncontrolled ROS production can damage all types of biological molecules (e.g., PUFAs, proteins, and mtDNA), inducing morphologic and functional alterations in mitochondrial membranes, with subsequent aggravation of oxidative stress. It was reported that the protective effects of Tau against nickel toxicity were related to improving mitochondrial function [159], as evidenced by the alleviation of LDH release into the plasma and the mitigation of disrupted mitochondrial membrane potential, reduced ATP production and decreased mtDNA content caused by nickel toxicity [159].

The mitochondrial membrane-stabilizing effects of Tau were also shown in an iron-overload murine model, as evidenced by the amelioration of mitochondrial swelling, the prevention of the loss of the mitochondrial membrane potential and the maintenance of the redox balance in the liver [55]. Membrane stabilisation could also be involved in the Tau-associated alleviation of the activities of AO enzymes, leading to improved AO defences and the regulation of neuroendocrine functions [160].

Our previous research with avian semen [161] showed that spermatozoa could be used as a valuable model to assess the membrane-stabilizing effects of various compounds. Spermatozoa membranes are known to contain high proportions of long-chain PUFAs, which are easily oxidised in stress conditions [162]. In common animal/poultry artificial insemination practices, easily measurable physiological (e.g., motility, cryo-resistance, etc.) and biochemical (e.g., enzyme release from spermatozoa into the medium/diluent) parameters of semen reflecting membrane stability and integrity are widely used. By adding test compounds into semen diluent, their membrane-modulating properties can be determined. For example, in rabbit [163] and canine [164] spermatozoa, Tau was found to inhibit membrane lipid peroxidation. The partial prevention of the osmotic stress-induced disintegration of plasma and outer acrosomal membranes of chimpanzee spermatozoa by Tau lead to the enhancement of cell viability [165]. During liquid storage (at 5 °C) of Mithun semen, Tau (50 mM) was reported to increase AO enzyme activity, reduce lipid peroxidation and prevent efflux of cholesterol from cell membranes [166]. Tau being included in the semen diluent was also reported to decrease damage to buffalo [167], buffalo and cattle [168,169], donkey [170], dog [171], chicken [172], ram [173,174,175], boar [176], stallion [177], rat [148,178], human [179], goldfish [180], and sea bass [181] spermatozoa.

### 7.4. Inhibition of Free-Radical Producing Enzymes by Tau

#### 7.4.1. Xanthine Oxidase

Xanthine oxidase (XO) catalyses purine degradation through the hydroxylation of hypoxanthine to xanthine, and further to uric acid, being an important source of ROS (e.g., O_2_^−^ and H_2_O_2_) and participating in inflammatory disease development [182,183]. Accumulating evidence indicates that Tau can ameliorate stress-induced increased XO activity. For example, in alloxan (ALX)-induced diabetic rats, Tau treatment decreased cardiac lipid peroxidation, protein carbonylation, and XO activity and ameliorated the activities of antioxidant enzymes [184]. Similarly, there was increased XO activity (71%) in diabetic rat kidney, and Tau administration prevented increasing XO activity, being an effective means of decreasing ROS production in diabetic kidney [185]. It was shown that KBrO_3_ increased XO in rat plasma more than 9-fold, and Tau was shown to have a significant protective effect by decreasing the elevated level of XO more than twice [186]. Tau was also found to reduce the elevated XO activities in serum of hyperuricemic rats [187]. The data presented above clearly indicate that Tau is able to decrease XO activity, and this action could be a valuable mechanism in understanding Tau AO activity in vitro and in vivo.

#### 7.4.2. NADPH Oxidase

NADPH oxidases (Nox) belong to a family of cytoplasmic enzymes participating in electron transfer across biological membranes. There are seven members of the Nox family of enzymes, namely Nox1, Nox2 (gp91phox), Nox3, Nox4, Nox5, Duox1 and Duox2 [188]. ROS generation by the NADPH oxidase enzyme complex is shown to play a vital role in various physiological processes, including the regulation of gene expression, the posttranslational modification of proteins, cell differentiation and immune host defence [189].

It seems likely that Tau affects NADPH oxidase activity by modulating NADPH content [190]. In fact, Li et al. [191] suggested that Tau can inhibit NADPH oxidase, a crucial source of cytosolic ROS in the norepinephrine-stimulated cardiomyocytes. The authors showed that Tau treatment of cardiomyocytes suppressed the activation of NADPH oxidase by norepinephrine and prevented ROS-induced activation of calpain and apoptosis. A substantial (20%) increase in NADPH oxidase activity was observed in the kidneys of alloxan-induced diabetic rabbits, and Tau was able to attenuate this phenomenon [192]. Miao et al. [193] also showed that Tau can down-regulate the expression of NADPH oxidase subunit p47phox in mammary tissues with *Streptococcus uberis*-induced mastitis. It was also suggested that the protection of Tau against ROS during N-methyl-D-aspartate (NMDA)-induced neuron injury is due to Nox inhibition [194]. Neuron cultures pre-treated with 25 mM Tau led to a lower percentage of cell apoptosis associated with reduced ROS level and ameliorated Nox2/Nox4 protein expression during NMDA-induced neuron injury. In addition, Tau also demonstrated Nox inhibition and neuroprotection against H_2_O_2_-induced injury [194]. Tau was found to suppress NADPH oxidase and attenuate ER stress, autophagy, and apoptosis in ARPE-19 cells [195], and to inhibit the paraquat and maneb-induced activation of NADPH oxidase (Nox2) in mouse microglial cells by interfering with the NF-κB pathway [196,197]. It seems likely that the suppression of NADPH oxidase-induced calpain activation by Tau could be a crucial mechanism of the anti-apoptotic effects of this amino-acid, offering new insights into the signalling mechanisms involved in taurine’s protective effects [198].

From the data presented above, it is possible to conclude that Tau is a vital regulator of NADPH oxidase activity, and this could be very relevant to various stress conditions when increased activity of the enzyme could cause overproduction of ROS and impose oxidative stress. It could well be that Tau is a crucial protective element for phagocyte cells, enabling them to prevent ROS overproduction and maintain their adequate antioxidant protection.

### 7.5. Antioxidant Protective Properties of Tau in Prevention of Toxic Effects of Various Chemicals

To address the antioxidant actions of Tau, it is necessary to choose proper model systems which would provide conclusive evidence related to potential molecular mechanisms of the protective action of the compound. Our idea was to use data accumulated when a search for effective antioxidants to decrease the toxic effect of various commonly used chemicals was conducted. The choice of toxic compounds of interest was associated with their proven prooxidant properties. Therefore, first of all, we chose data on arsenic (As) and carbon tetrachloride (CCl_4_), both of which are important human health hazards. Secondly, we used thioacetamide (TAA) and fluoride, chemicals possessing hepatotoxicity. Thirdly, we chose cisplatin (CP), doxorubicin (DOX) and streptozotocin (STZ), chemotherapy drugs with substantial side effects, including nephrotoxicity, cardiotoxicity and diabetes promotion. All of the aforementioned chemicals were clearly proven to impose oxidative stress by means of the downregulation of various parts of the antioxidant defence network. Finally, we looked at other toxic elements with proven prooxidant activities [199,200]. In general, accumulating evidence confirms the importance of oxidative stress as an important mechanism of chemical hepatotoxicity [201]. Therefore, the toxic compounds were employed in a variety of experimental models to study the antioxidant properties of Tau. It has been shown that Tau can protect cultured cells, organs and mammalian species against the detrimental consequences of oxidative stress imposed by various chemical agents, biochemical, drugs, toxins and disease states. A summary of recent findings is presented below.

#### 7.5.1. Arsenic

Arsenic (As) is a toxic and carcinogenic element, representing a serious risk to human health worldwide [202]. As-induced toxicity is shown to lead to elevated ROS production, oxidative stress and increased lipid peroxidation, protein carbonylation and DNA oxidation, as well as disrupted cell signalling, resulting in apoptosis [203]. The toxic effect of As was associated with significantly reduced activities of major AO enzymes (SOD, GPx, GST, GR, CAT) and decreased concentrations of non-enzymatic antioxidants (vitamins C and E, GSH) in the liver [204], heart [205], kidney [206], brain [207], testes [208] and pancreas [209] of the experimental animals.

In As-induced oxidative stress and toxicity, various antioxidants are shown to attenuate the aforementioned detrimental changes in the antioxidant defence network. In particular, the protective antioxidant effects of Tau against As toxicity in vitro and in vivo have been widely observed. For instance, As treatment caused oxidative stress associated with a reduction in the activities of the AO enzymes, including SOD, GPx, CAT, GST and GR. The same treatment was shown to reduce the concentration of GSH, increase the level of GSSG and induce lipid peroxidation. However, adding Tau to NaAsO_2_-treated hepatocytes was reported to improve antioxidant defences and ameliorate lipid peroxidation [210]. Arsenic was shown to reduce cardiomyocyte viability, imposing oxidative stress associated with increased ROS production and intracellular calcium overload. Enhanced apoptotic cell death through mitochondria-dependent caspase-3 activation and poly-ADP ribose polymerase cleavage was also observed. Tau ameliorated NF-κB activation via the IKK, p38 and JNK MAPK signalling pathways, and partly prevented As-induced myocardial damage, being an effective protectant against As-induced cardiovascular burden [205].

The administration of Tau significantly reduced oxidative stress and As-induced oxidative injury in rat livers [204]. In addition, it was shown that Tau can alleviate As_2_O_3_ induced liver inflammation by suppressing the autophagic-CTSB-NLRP3 inflammasomal pathway [211]. Furthermore, Tau administration was found to ameliorate As-induced insulin resistance as a result of the activation of PPARγ-mTORC2 signalling and the inhibition of hepatic autophagy in mice [212]. Treatment with Tau prior to As administration was demonstrated to mitigate As-induced oxidative renal dysfunction and apoptosis in rats [206], and Tau also had a protective effect against As-induced DNA damage in mice kidneys [213]. There are also other mechanisms of the protective effects of Tau against As toxicity, including restoration of the disturbed mineral balance. For example, Tau was reported to normalise the levels of Se, Cu, and Fe in the liver and kidneys of mouse exposed to As [214]. Taking into account the important roles of the mentioned elements as vital co-factors of antioxidant enzymes (e.g., Se is a part of 25 selenoproteins, Cu is involved in SOD and Fe is a part of CAT), it is possible to explain the protective effects of Tau on AO enzymes in As intoxication. Oral administration of Tau in the same dose was reported to be effective in counteracting As-associated oxidative stress, attenuating apoptosis in testicular tissue and maintaining testicular integrity through the regulation of NF-κB, phospho-ERK1/2, phospho-p38, phospho-Akt and Bcl-2/Bad [208].

It is well known that brain lipids contain high levels of PUFAs, making the tissue very susceptible to oxidative damage. Therefore, the activities of the antioxidant enzymes as well as the levels of GSH and total thiols in rat brain have been reported to be significantly reduced due to As exposure. At the same time, oral administration of Tau (100 mg/kg/body weight for 5 days) was shown to effectively prevent As-induced oxidative stress in the brain tissue of the experimental rats [207]. It was also shown that Tau can alleviate DNA damage of mouse brain neurons caused by As through the RNS signal pathway [215]. Similarly, in the mouse cerebrum, As exposure induced lipid peroxidation and a decrease in Nrf2 expression, and these changes were ameliorated by Tau treatment [216]. Tau was indicated to mitigate the down-regulated expression of thyroid hormone receptor in the brains of As-exposed mice [217]. As a result of the improvement of antioxidant defences, Tau supplementation significantly ameliorated As-induced apoptosis through the mitochondria pathway in mouse hippocampus [218]. Dietary Tau (150 mg/kg) was also found to ameliorate decreased GSH concentration and increased lipid peroxidation (MDA) in the liver of As-exposed mice [219].

It seems likely that inhibition of the Nrf2/Trx pathway plays a crucial role in the pathogenesis of As-induced oxidative stress and diabetes. For example, after treatment with As_2_O_3_, the expression of the Nrf2 protein and Trx gene were significantly decreased in the offspring’s pancreas, associated with increased ROS generation and stimulation of autophagy in As-treated pancreas. Tau was shown to reverse As-inhibited Nrf2 and Trx and inhibit autophagy [209]. Interestingly, Tau can decrease oxidative stress and rescue the As-induced injury not only in cell culture (INS-1 cells), but also in the pancreas of rat offspring [220].

The model system based on the As-treatment of experimental animals clearly indicated that Tau was effective in counteracting oxidative stress. Tau administration showed a significant protective effect on the antioxidant defence network by maintaining the activity of AO enzymes and the effective concentrations of non-enzymatic antioxidants in As-treated rats. Therefore, Tau can mitigate As-induced oxidative stress and renal damage as a result of its antioxidant protective activity and involvement in the regulation of MAPKs/NF-κB and mitochondria-dependent pathways.

#### 7.5.2. Carbon Tetrachloride

Carbone tetrachloride (CCl_4_) is an industrial solvent and refrigerant production intermediate possessing high toxicity and causing severe oxidative stress in the liver [221]. The metabolism of CCl_4_ via CYP2E1 with the production of ROS is an important mechanism of its toxicity [222]. In particular, metabonomic analysis revealed that CCl_4_ exposure leads to oxidative stresses in various rat organs, altering their functions [223]. In general, CCl_4_ can compromise antioxidant defences by inhibiting AO enzymes (SOD, GPx and CAT) and decreasing GSH in liver samples [224], leading to oxidative stress and hepatic injuries [225] with the enhancement of lipid peroxidation in the liver [226] and serum [227].

In 1982, it was suggested that hepatic Tau could play a protective role in hepatocytes against hepatotoxicants such as CCl_4_, and that dietary Tau may be a useful treatment in hepatotoxin-induced liver injury [228]. However, subsequently, in a paper from the same department, it was shown that pre-treatment with Tau facilitated hepatic lipid peroxide formation associated with CCl_4_ intoxication in rats [229]. It seems likely that Tau’s effect on CCl_4_-treated animals is condition-dependent, since later studies clearly showed the protective effect of Tau in CCl_4_ toxicity. First of all, the results of in vitro studies suggest that Tau protects cells against toxic damage. For example, hepatocytes were incubated with various concentrations of CCl_4_, and other toxicants, namely hydrazine and 1,4-naphthoquinone, in the presence and absence of Tau (0–15 mM). The presence of Tau decreased the cytotoxicity of each compound, as indicated by trypan blue uptake and LDH leakage. Importantly, the protection was concentration-dependent, with a significant effect at 10 mM for all three compounds [230]. Later, it was confirmed that Tau protected isolated hepatocytes against CCl_4_ and hydrazine and 1,4-naphthoquinone cytotoxicity [231]. In isolated rat hepatocytes, CCl_4_ induced LDH release and decreased cellular thiols, causing oxidative stress. Treating with Tau ameliorated these changes [232]. Pre-treatment of cultured neurons with Tau was shown to mitigate the loss of GPx activity and decrease lipid peroxidation caused by CCl_4_ treatment. Furthermore, in CCl_4_-intoxicated mice, Tau was able to improve antioxidant defences by inducing the GPx activity in a dose-dependent manner in the brain [233].

Interestingly, CCl_4_ can affect Tau assimilation and metabolism, as reflected by the elevation of urinary Tau levels in rats. It seems likely that Tau synthesis is compromised in oxidative stress conditions due to the depletion of GSH [234]. Earlier, the authors suggested that Tau synthesis in the liver is increased in response to a toxic insult, and subsequent leakage from damaged cells could lead to increased Tau levels in the urine [235]. The confirmation of the special protective role of Tau in CCl_4_ toxicity came from a study where a reduction in liver Tau in rats caused by beta-alanine treatment was associated with increased CCl_4_ toxicity [236]. Further confirmation of the protective effect of Tau in CCl_4_ toxicity came from Tau-deficient animals. Depletion of Tau in rats was shown to increase susceptibility to liver damage from CCl_4_, and susceptibility to a variety of hepatotoxicants was found to correlate with the hepatic Tau concentration [237]. Damage to liver DNA and the abnormal expression of the hepatic extracellular matrix were observed in the CCl_4_-treated rats, and Tau partly prevented DNA damage, necrosis and hepatocellular degeneration induced by CCl_4_ [237]. The increase in hepatic lipid peroxidation in the CCl_4_-exposed rats was mitigated by Tau pre-treatment, but Tau did not affect GSH and hydroxyproline content in the CCl_4_-treated rats. However, Tau pre-treatment decreased hepatocellular necrosis and atrophy [238]. Similarly, it was shown that Tau can decrease liver damage (serum transaminase activities and hepatic lipid peroxidation) without changing the hepatic antioxidant system in rats with hepatic fibrosis caused by the treatment of rats with ethanol and CCl_4_ [239]. Oral Tau administration increased hepatic Tau accumulation, reduced oxidative stress and ameliorated hepatic fibrosis in CCl_4_-treated rats [240]. Similarly, Tau treatment significantly reduced fibrosis scores and organelle injury scores in CCl4-treated rats [241]. The liver of the Tau-treated rats was protected against fibrosis and oxidative stress (LPO and 8-OHdG) caused by CCl_4_ treatment. Proliferation, oxidative stress, and fibrogenesis were significantly inhibited in hepatic stellate cells by treatment with Tau [242]. In addition, Tau administration (100 mg/kg b.w. twice weekly for 4 weeks) was shown to ameliorate CCl_4_-induced reduction in CAT activity and GSH concentration and to prevent lipid peroxidation (MDA) in the testicular tissues of CCl_4_-administered male Wistar rats [243].

It seems likely that Tau may act in concert with other antioxidants, being a valuable part of the antioxidant defence network. For example, recently, the hepatoprotective effects of Tau alone or in combination with silymarin (SIL) on CCl_4_-induced liver damage were investigated. It was shown that CCl_4_ caused substantial increases in lipid peroxidation in the liver associated with oxidative stress leading to significant declining activities of SOD, GPx, GR, GST and GSH concentrations. The expression of serum pro-inflammatory and fibrogenic cytokines including TNF-α, TGF-β1, IL-6, leptin and resistin was shown to be induced, while the level of anti-inflammatory (adiponectin) cytokine was suppressed in experimental rats. The obtained results also show that CCl_4_ induced liver injury, as shown by increased serum ALT, AST, ALP, GGT and bilirubin levels. Interestingly, both Tau and SIL post-treatments relieved most of the above-mentioned imbalances. However, the combination of two compounds with antioxidant properties was shown to be more effective than single applications in reducing oxidative stress due to CCl_4_ treatment. Furthermore, the protective effects of Tau and SIL were confirmed by histological and ultrastructural assays [244]. Furthermore, a proteomic analysis of the results of a combination therapy (Tau + epigallocatechin gallate + genistein) of CCl_4_-treated rats showed that antioxidant defences of the liver were restored, and this led to antifibrotic action of the antioxidant mixture [245].

In the aforementioned studies, Tau treatment was able to restore the major parameters of the antioxidant defence network in the liver which were initially compromised by CCl_4_ treatment. Therefore, tissue injuries were significantly ameliorated. Thus, the second model based on the prooxidant properties of CCl_4_ in animals clearly confirmed antioxidant action of Tau associated with the restoration of the antioxidant enzyme activities and redox balance, leading to the prevention of/decrease in the detrimental consequences of the CCl_4_-associated oxidative stress

#### 7.5.3. Thioacetamide

Thioacetamide (TAA) is a potent hepatotoxicant, and in the development of TAA-induced toxicity, ROS formation and oxidative stress are thought to play a crucial role [199,246]. In particular, TAA-induced cirrhosis is associated with oxidative stress in the liver, which is associated with a high level of lipid peroxidation, accompanied by distorted AO status and redox balance [247]. Therefore, TAA-treated animals were used to evaluate the antioxidant protective effects of Tau. It was shown that Tau protected animals against TAA-induced liver cirrhosis by decreasing oxidative stress. TAA treatment caused oxidative stress, as indicated by increased lipid peroxidation (MDA and diene conjugates) and decreased levels of cellular antioxidants, including GSH, vitamin E, and vitamin C, alongside reduced activities of GPx in the rat liver. When Tau (2% *w*/*w*) was administered together with TAA for 3 months, a reduction in TAA-induced hepatic lipid peroxidation and increase in TAA-depleted vitamin E levels and GPx activities were observed. The protective effects of Tau on TAA-induced hepatic cirrhosis were also confirmed by histopathological findings [248]. In another study, Tau administration (400 mg/kg, i.p., every 12 h and started 24 h prior to the first TAA injection) was found to decrease liver damage, as indicated by reduced serum transaminase activities and decreased hepatic lipid peroxidation. However, the studied indexes of the antioxidant systems (e.g., vitamins E, C, SOD and GPx) were not affected [249]. It seems likely that the protective effect of Tau on antioxidant enzymes compromised due to TAA treatment depends on the experimental conditions. For example, in a zebrafish model, TAA injection induced steatosis associated with increased lipid peroxidation, and Tau attenuated these changes. Importantly, SOD activity in the Tau-TAA group was significantly increased in comparison to the TAA group. Furthermore, the mRNA expression of the vitagene SIRT1 (0.5-fold) and adiponectin receptor 2 (0.39-fold) were lower in the TAA group than the control, while TNF-α mRNA expression was 6.4-fold higher in the TAA group than the control. In this case, protective effects of Tau were also evident: SIRT1 mRNA expression was 2.6-fold higher in the Tau-TAA group than in the TAA group [250]. Tau is shown to have a potential protective role against hepatic encephalopathy and hyperammonaemia-induced mitochondrial dysfunction. For example, brain and liver mitochondrial function in the TAA-treated rats was compromised, as indicated by increased ROS production, suppressed succinate dehydrogenase activity, mitochondrial swelling, collapsed mitochondrial membrane potential and decreased ATP concentration. Tau treatment (250, 500, and 1000 mg/kg BW) was shown to decrease mitochondrial swelling, ROS, and LPO and restore brain and liver mitochondrial ATP [251]. Tau (100 mg/kg, IP, daily) was demonstrated to ameliorate TAA-induced liver fibrosis in rats via the improvement of AO defences (GSH and MDA) and the modulation of the Toll-like receptor 4/NF-κB signalling pathway [252]. In rats, Tau in the same dose also showed reno-protective properties in TAA-induced kidney injury through its antioxidant (Nrf-2 induction and activation of HO-1 and NQO-1) and anti-inflammatory (suppression of NF-κB transcription and inhibiting the production of pro-inflammatory mediators) actions [253].

The aforementioned data on the protective effect of Tau in TAA-treated animals confirmed the antioxidant action of this β-amino acid associated with the restoration of the antioxidant enzyme activities and vitagene activities compromised by the toxicant. This led to decreased lipid peroxidation, mitochondria stabilisation and the prevention of other detrimental consequences of oxidative stress.

#### 7.5.4. Cisplatin

Cisplatin (CP) is the most commonly used chemotherapy drug for the treatment of various cancers, and its nephrotoxicity is thought to be associated with ROS overproduction [254], decreasing the activities of AO enzymes (SOD and GPx) and GSH content, as well as increasing lipid peroxidation in rat livers [255,256]. CP can also cause structural damage to the liver, as indicated by the significantly elevated serum activities of LDH and creatine kinase [256].

Therefore, it is proven that oxidative stress plays an important role in CP-induced renal damage, and several dietary antioxidants have been shown to ameliorate this detrimental effect [257]. It was shown that Tau can effectively attenuate the deleterious effect of CP on the renal tubular function. Tau supplementation (1.5% Tau in the drinking water) restored the kidney GSH content and GPx activity, leading to a reduction in lipid peroxidation in the kidney tissue [258]. It seems likely that oxidative stress and inflammation are major players in the pathogenesis of CP-induced acute renal failure and Tau can suppress oxidative stress and inflammation in CP-exposed animals. For example, treatment with Tau (IP in a dose of 50 mg/kg BW, 3 times weekly for 8 weeks) after CP administration was reported to ameliorate CP-induced nephritic oxidative stress markers (kidney and serum MDA and total serum AOA) and nephritic inflammation as compared with the CP-treated group [259]. In addition, Tau (25 mM) pre-treatment rescued CP-induced muscle (C2C12 myotubes) atrophy in vitro [260]. Recently, it was shown that Tau can provide protection against CP-induced cardiac damage by modulating inflammatory responses and oxidative stress. CP exposure led to the upregulation of the unfolded protein response (UPR)-regulated CCAAT/enhancer binding protein, the induction of GRP78 (a marker of ER stress), eIF2α signalling and apoptosis. In the same experiment, Tau administration (150 mg/kg body wt., i.p.) significantly ameliorated the ROS production, mitigated the overexpression of NF-κB, and suppressed the elevation of pro-inflammatory cytokines, adhesion molecules, and chemokines due to CP exposure [261]. CP was shown to enhance lipid peroxidation and inhibited the major AO enzymes (SOD, GPx, GR and CAT) in rat kidney tissue, while treatment with Tau was able to protect against the detrimental changes in the serum, urine, and renal tissue of CP-treated animals [262]. Interestingly, combined therapy of Tau and N-acetylcysteine was more effective in ameliorating CP-induced nephrotoxicity, reflecting their synergistic effect. Rats pre-treated with taurine (100 and 200 mg/kg BW) and exposed to CP showed significant improvements in brain antioxidant defences, as evidenced by improved SOD, CAT, and GST activities and GSH concentration with concomitant decrease in lipid peroxidation in the brain when compared with the CP alone group [263].

It could well be that the negative effects of CP on the antioxidant defences are mediated via a decreased Tau status. For instance, CP was shown to down-regulate *TauT* expression through the p53-dependent pathway. In fact, CP increased the p53 expression, which, in turn, repressed *TauT* [264]. At the same time, overexpression of *TauT* was found to protect against CP-induced kidney injury in a *TauT* transgenic mouse model [265]. The expression of *TauT* was shown to be negatively regulated by p53 and positively regulated by c-Jun [43,266]. Interestingly, CP induced the apoptosis of LLC-PK1 cells in a dose-dependent manner, while forced over-expression of *TauT* in LLC-PK1 cells was able to attenuate CP-induced down-regulation of Tau uptake by LLC-PK1 cells and prevented apoptosis in renal tubular cells [264]. Detrimental changes in the redox status of cells, *TauT* down-regulation and p53 up-regulation in renal tissues of rats due to CP injection were shown to cause oxidative stress associated with increased 8-OHdG expression, an indicator of oxidative DNA damage. Tau administration prior to a CP injection was demonstrated to abrogate the decline in antioxidant defences and to inhibit the elevation in DNA damage and inflammation [267]. Similarly, Tau prevented the formation of CP-related DNA lesions caused by iNOS-mediated nitrative stress [268]. Tau intake (50, 150, and 250 mg/kg on alternate days followed by CP) was shown to ameliorate CP-induced testicular damage in rats due to the restoration of AO defences (increased GSH and reduced MDA) and anti-apoptotic effects (decreased Bax, increased bcl2; [269]). Tau was also demonstrated to promote GSH biosynthesis and facilitate ROS clearance in CP-treated myoblasts [270].

Considering CP treatment as a model for studying the antioxidant actions of Tau, it is possible to conclude that:

- Tau is able to abrogate decreased activities of antioxidant enzymes (SOD, GPx, GR, and catalase) and non-enzymatic antioxidants (GSH) in CP-treated animals and maintain optimal redox status in stress conditions;
- Oxidative stress imposed by CP treatment was associated with DNA oxidation and apoptosis, and Tau was shown to be protective;
- CP treatment affected Tau metabolism and transport, while the overexpression of *TauT* in CP-treated animals showed protective effects;
- Tau can ameliorate increased NF-κB expression caused by CP-treatment and prevent excessive inflammation.

#### 7.5.5. Doxorubicin

Doxorubicin (DOX, also called adriamycin) is a drug widely used for the treatment of cancer patients. However, DOX’s side effects are related to cardiotoxicity, associated with oxidative stress, redox disbalance, mitochondrial dysfunction and apoptosis [271]. For example, in rat cardiomyocytes, DOX treatment significantly elevated cellular ROS and decreased the levels of Tau, Akt, and phosphorylated Akt and Bad [272]. In general, the molecular mechanisms of DOX’s cardiotoxicity have not yet been described, but impairment of the antioxidant defence system and oxidative stress associated with cell membrane damage and mitochondrial dysfunction have been considered as important etiologic factors [273].

In 1988, the effect of Tau on DOX-induced cardiotoxicity was examined in mice [274]. A single intraperitoneal injection of DOX induced oxidative stress, as indicated by decreased GPx activity and increased lipid peroxidation in the mouse myocardium. The combined oral and intraperitoneal administration of Tau was shown to decrease lipid peroxidation and improved the survival rate of the mice treated with DOX [274]. Ten years later, it was confirmed that Tau (3% *w*/*v* in water) can prevent oxidative stress by suppressing ROS generation and restoring the physiological level of GSH in the heart of DOX-treated mice [275]. This was associated with a significantly reduced mortality rate of DOX-intoxicated mice. At the next stage of research devoted to oxidative stress as the leading mechanism of DOX toxicity, an in vitro study was conducted. Increased ROS generation, collapsed mitochondrial membrane potential, DNA fragmentation, and apoptosis in primary cultured neonatal rat cardiomyocytes were observed as a result of DOX treatment [276]. Furthermore, DOX increased p53, JNK, p38 and NF-κB phosphorylation, disturbed the Bcl-2 family protein balance and activated caspase 12, caspase 9 and caspase 3. Again, Tau treatment alleviated all of the adverse effects of DOX. The authors showed that the mechanism of Tau-induced cardioprotection is associated with the activation of specific survival signals and PI3-K/Akt, as well as the suppression of p53, JNK, p38 and NF-κB [276]. Similarly, in vitro Tau treatment attenuated DOX-induced cytotoxicity and suppressed ROS formation in B16F10 cells [277].

Furthermore, the protective effect of Tau on DOX-treated animals was observed in vivo. In particular, in DOX-treated rats, Tau was shown to attenuate disturbances of the antioxidant defence system of testes and prevent oxidative stress by maintaining the GSH level and the activities of SOD, CAT, GST, GPx and GR. Tau was indicated to be able to prevent nearly all of the aforementioned DOX-induced testicular abnormalities [278]. Similarly, in rats treated with DOX, Tau zinc was able to dose-dependently increase the liver SOD activity and GSH concentration, and reduced the MDA level [279]. It seems likely that by suppressing oxidative stress and apoptosis in DOX-treated animals, Tau can prevent acute hepatic damage. For example, Tau treatment (10 mg/kg BW) was shown to prevent oxidative stress by maintaining the SOD activity and GSH content in the liver of DOX-exposed mice. Furthermore, Tau attenuated the increased expression of mRNAs for Fas and Bax after DOX treatment [280]. DOX-induced acute hepatic damage was markedly prevented by Tau. Similarly, in the kidneys of mice with DOX-induced renal injury, the augmented expression of inflammation-related mRNAs, including NF-κB, COX-2, and iNOS, was down-regulated by Tau treatment (50 and 100 mg/kg BW for 5 days [281]). Tau supplementation was found to have protective effects against the myocardial damage caused by DOX in mice by improving antioxidant capacity (increased GSH content, SOD activity and GPx4 expression) and decreasing oxidative damage and apoptosis [282]. It is important to mention that similarly to the action of other toxicants (e.g., CP exposure), DOX-treatment was associated with reduced expression of the *TauT* gene in cultured cardiomyocytes obtained from neonatal rat hearts [283]. Interestingly, knockdown of *TauT* impaired human embryonic kidney development, due to changes in the expression of cell cycle-related genes [284].

Data on oxidative stress caused by DOX treatment of laboratory animals and the protective effects of natural antioxidants also confirmed important antioxidant actions of Tau:

- DOX compromised the antioxidant defence system by decreasing antioxidant enzyme (SOD, CAT, GST, GPx and GR) activities and non-enzymatic (GSH) antioxidant concentrations, and Tau treatment was able to ameliorate these detrimental changes;
- the mechanisms of Tau-induced cytoprotection were suggested to be also associated with the activation of specific survival signals and PI3-K/Akt, as well as the suppression of p53, JNK, p38 and NF-κB.

#### 7.5.6. Streptozotocin

Streptozotocin (STZ) is an antineoplastic agent used to treat pancreatic carcinoma and to induce diabetes in experimental animals by causing specific necrosis of the pancreatic β-cells [285]. At the same time, STZ was shown to cause oxidative stress, as evidenced by accelerated hypothalamic lipid peroxidation, an increased protein carbonyl content and a reduction in GPx activity and GSH content in parallel with mitochondrial impairment, marked by an increase in mitochondrial membrane permeabilisation [286].

The main research on the protective effects of Tau against oxidative stress imposed by STZ was conducted with brains and testes, tissues which are rich in PUFAs and are most sensitive to lipid peroxidation [287]. For example, Tau has been reported to improve STZ-induced diabetes mellitus associated with oxidative stress. Tau treatment restored Tau level, decreased lipid peroxidation (MDA plus 4-hydroxyalkenals) and restored ascorbic acid levels in the sciatic nerves of diabetic rats [288]. Furthermore, Tau administration to STZ-treated rats was shown to preserve/protect antioxidant enzyme (GPx, GR, GST, CAT and SOD) activities in the hippocampus [289]. In a later, more detailed study, significant detrimental changes in the brain AO defence system were observed due to STZ treatment: raised MDA (+59%), lowered GSH/GSSG ratio (−46%), and decreased activities of CAT (−43%), GPx (−48%), SOD (−65%), GR (−50%) and GST (−51%). However, Tau was shown to significantly ameliorate the aforementioned detrimental changes in the antioxidant defence system of the rat brain caused by STZ [290]. In addition, antioxidant (increased SOD activity and GSH content and decreased MDA level) and anti-inflammatory (decreased TNF-α and IL-1β expression) activities of Tau in the cortex and hippocampus of STZ-treated rats were observed [291]. Recently, the effects of Tau on oxidative stress, DNA damage and inflammation in the frontal cortex and hippocampus of STZ-induced diabetic rats were evaluated. As expected, diabetic rats were characterised by oxidative stress in the brain, as evidenced by increased levels of ROS and DNA damage and increased levels of inflammatory mediators (IL-6, IL-12, TNF-γ, and IFN-α). Tau treatment was shown to significantly reduce oxidative stress, leading to reduced ROS, DNA damage, and inflammatory cytokine levels [292].

Tau treatment decreased diabetes-induced oxidative stress in the testes, as evidenced by decreased MDA and 8-OH-dG levels and increased CAT activity, inhibited apoptosis and decreased morphological damage in the testicular tissue of rats [293]. Similarly, Tau significantly reduced lipid peroxidation, DNA damage, inflammation and oxidative stress by enhancing the activity of the antioxidant enzymes in the testes of STZ-induced diabetic rats [294]. Furthermore, rat exposure to STZ imposed testicular ER stress, caused the translocation of NF-κB to the nucleus, and activated the mitochondria-dependent apoptotic pathway and DNA fragmentation. In such conditions, administration of Tau (100 mg/kg body weight for 6 weeks post diabetic induction) was shown to successfully ameliorate all of the aforementioned adverse effects [295]. Interestingly, Tau treatment was also found to recover testicular steroidogenesis and spermatogenesis in STZ-induced diabetic rats [296].

Similar antioxidant protective effects of Tau were observed in other tissues. For instance, oxidative stress in SRZ-induced diabetic rats was evidenced by increased renal levels of MDA (+42%), decreased renal GSH redox state (−71%), and reduced activities of catalase (−70%), GPx (−71%) and SOD (−85%). Tau provided significant protection against detrimental changes in the GSH redox state and in SOD, GPx and CAT activities, which were compromised due to STZ treatment [297]. Dietary Tau supplementation during pregnancy was shown to provide significant protection against diabetes-associated oxidative stress in the liver, both in mothers and embryos [298]. Tau was indicated to be effective in attenuating the AO system alterations (decreased SOD, GPx and GSH) brought about by diabetes in both the aorta and heart of rats [299]. Similarly, Tau was shown to inhibit MDA formation and prevent changes in the redox status and oxidative stress in both the plasma and RBC of diabetic rats [300]. Pre-treating STZ-induced diabetic rats with Tau (by the i.p. route in two equal doses, 1.2 mM/kg each at 75 and 45 min before STZ) was reported to protect the brain against oxidative stress by improving AO defences, as indicated by the restoration of GSH content as well as CAT, GPx and SOD activities [301].

Comprehensive data on STZ-induced oxidative stress in various animal tissues indicate that this toxic agent can be used in a model system to study the antioxidant protective effects of Tau. In fact, the obtained data can be summarised as follows:

- antioxidant enzyme activities (SOD, GPx, GR, GST, Catalase) in animal tissues (brain, testes, kidney, liver, aorta, heart, plasma and RBC) are shown to be compromised by STZ treatment;
- Tau supplementation of STZ-treated animals in most cases can prevent oxidative stress through the restoration of antioxidant enzyme activities and redox balance in cells/tissues, leading to a reduction in lipid and DNA oxidation;
- Tau also showed anti-inflammatory action in STZ-treated animals, probably due to decreasing NF-κB expression in various tissues.

#### 7.5.7. Fluoride

Fluoride is an important industrial chemical used in various areas of human life. It is generally accepted that fluoride exposure can cause tissue damage, due to oxidative stress associated with compromised AO defences and the breakdown of redox balance [302]. Furthermore, the proteomic analysis indicated important changes in proteins related to cellular respiration, mitochondrial oxidative stress, the endoplasmic reticulum and apoptosis upon exposure to fluoride [303].

The cytoprotective effect of taurine on fluoride-induced oxidative stress and apoptosis in murine hepatocytes in vitro has been clearly demonstrated [304]. Later, it was shown that Tau (100 and 200 mg/kg BW) ameliorated fluoride-induced renal and thyroid dysfunctions in rats due to the reduction in oxidative stress (decreased H_2_O_2_ and MDA levels, increased SOD and CAT), and enhancement of the functional status of the thyroid system [305]. In a study from the same department, the levels of H_2_O_2_ and MDA were significantly increased, whereas SOD and CAT activities were reduced in the hypothalamus, cerebrum and cerebellum of NaF-treated rats [306]. The administration of Tau (100 and 200 mg/kg BW) enhanced SOD and CAT activities, as well as decreasing H_2_O_2_ and MDA levels in the NaF-exposed rats [306]. Therefore, the neuroprotective mechanism of Tau against NaF-induced brain toxicity is likely due to its ability to enhance antioxidant defence system and subsequently inhibit oxidative stress in the treated animals. Interestingly, Tau significantly prevented NaF-induced oxidative stress, augmented antioxidant activities and increased GSH level in the brain, testes, and epididymis of the treated rats [307]. Therefore, similarly to its protective effects against other toxicants, in the case of NaF treatment, Tau provides antioxidant protection via maintaining antioxidant enzyme activities and cell/tissue redox balance, leading to the prevention of oxidative stress and damage to lipids and DNA.

#### 7.5.8. Other Toxicants

It was shown that Tau exerted AO protection against toxicity induced by heavy metals including cadmium [308,309,310,311,312], mercury [313], hexavalent chromium [314], nickel [159,315], aluminium [316,317], lead [318], chlorpyrifos plus lead [319] and iron-overload [55,320]. The protective effect of Tau was also seen against the toxicity of cancer/chemotherapy drugs, including methotrexate [321], tamoxifen [141,322], methotrexate [323], 5-fluorouracil [324], and cyclophosphamide [325], as well as against immunosuppressive and immunomodulating compounds such as cyclosporine A [326], 7,12-dimethyl benz[a]anthracene [327], endotoxin [328] and LPS [329]. In addition, Tau was able to protect the AO system against toxicity imposed by various clinically relevant drugs, including amodiaquine [330], sulfasalazine [331], methimazole [332], alendronate [333], isoproterenol [334], indomethacin [335], gentamicin [336], methamphetamine [337], nandrolone decanoate [338], acetaminophen [339], ibuprofen [340] and morphine [341].

The protective effects of Tau on the antioxidant systems of the body are also shown in pesticide toxicity (methiocarb; [342] and lambda-cyhalothrin: [343]) and insecticide-related toxicity (permethrin: [344], endosulfan: [345], dichlorvos: [346]). Tau showed high protective efficacy against typical prooxidants such as butyl hydroperoxide [347], peroxynitrite [348], H_2_O_2_ [349] and ozone [350]. Tau was proven to be protective against AO disturbances imposed by dietary D-galactose [351], a high-fructose diet [352], a high-cholesterol diet [353] or alloxan treatment [354]. Furthermore, the beneficial effect of Tau is shown to be universal and includes protection from toxicity caused by other chemicals, including nicotine [333], acrylonitrile [355], potassium bromate [186], diethylnitrosamine [356], triorthocresyl phosphate [357] and 3-nitropropionic acid [358].

In most of the aforementioned cases, the protective antioxidant effect of Tau against various toxicants was associated with the prevention of/decrease in oxidative stress imposed by toxic elements. This was associated with the restoration/maintenance of the activities of antioxidant enzymes and non-enzymatic antioxidants (GSH) responsible for the redox balance in cells/tissues.

Our analysis of published data related to the protective effects of Tau against toxicity of various chemicals, including As, CCl_4_, TAA, fluoride, CP, DOX, and STZ revealed several common mechanisms. Antioxidant enzyme activities were used as the main indexes of the antioxidant defence system:

- SOD—main adaptive enzyme of the first level of antioxidant defence, participating in the detoxification of superoxide radicals. It is regulated at the vitagene level [359];
- GPx—a family of AO enzymes of the first level of antioxidant defence, converting toxic H_2_O_2_ into water, an essential step in the completion of superoxide radical detoxification [360,361,362];
- GSH—an endogenous antioxidant tripeptide considered as one of the most important cellular antioxidants. GSH can directly detoxify ROS/RNS or contribute indirectly to AO defences as a cofactor for various antioxidant enzymes, such as GPx and GST, participating in maintaining redox balance in various tissues of the body [1];
- GR—an essential component of the glutathione system responsible for recycling oxidised glutathione back into the reduced form, involved in redox balance regulation [1];
- GSTs—a superfamily of multifunctional isoenzymes participating in the cellular detoxification of various endogenous and exogenous toxic compounds, including insecticides, drugs, and organic hydroperoxides. GSTs play a crucial role in protection against various types of cellular oxidative damage [363];
- CAT—an important enzyme of the first level of the antioxidant defence network, providing detoxification of H_2_O_2_ produced by SOD [364].

The results of the aforementioned studies indicate the following. First of all, Tau supplementation can prevent oxidative stress by restoring antioxidant enzyme activities (SOD, GPx, GR, GST, CAT) and non-enzymatic antioxidants (GSH, ascorbic acid) compromised by toxicant treatment. It is well-established that antioxidant enzymes belong to the first level of antioxidant defence and, in many cases in stress conditions, they are inducible [1]. However, when the stress level is too high, their activities usually decrease. This was the case in most of the aforementioned studies with toxic chemicals. GSH is known to play a crucial role in cellular redox balance regulation. Indeed, in most analysed cases, toxic elements reduced the GSH/GSSG ratio, reflecting important disturbances in the redox status. It could well be that the protective effect of Tau is associated with activation transcription factor Nrf2, responsible for the additional synthesis of antioxidant enzymes and non-enzymatic antioxidants.

**Figure 1:**
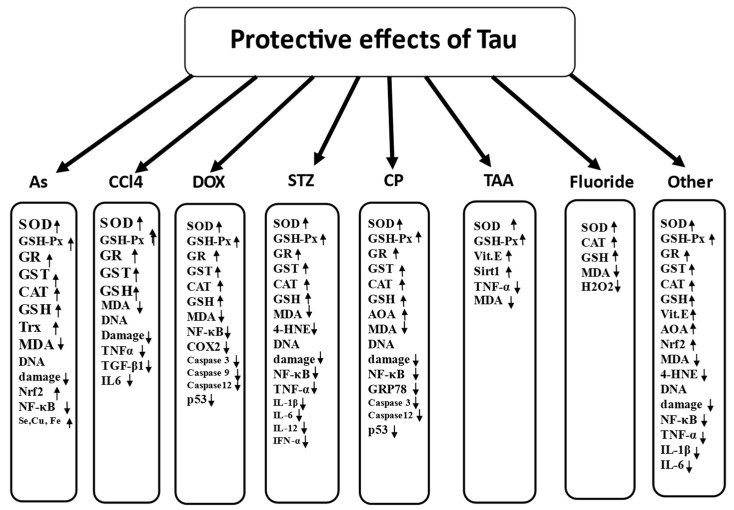
Protective antioxidant effects of Tau. For abbreviations, refer to the index (adapted from [1]).

Additional mechanisms of antioxidant enzyme activity restoration could be related to the ability of Tau to normalise the levels of Se, Cu, and Fe in the tissues of animals exposed to toxicants. Tau was shown to restore the redox status of the cells/tissues. Interestingly, the protective effect of Tau was observed in vitro in cell cultures and in vivo in various tissues, including the brain, testes, kidneys and liver. Secondly, the main indexes of lipid peroxidation (MDA and 4-hydroxyalkenals) and DNA oxidation (8-OHdG) were also retuned to physiological levels after Tau treatment. Thirdly, Tau treatment alleviated the DOX-induced increase in p53, JNK, p38 and NF-κB phosphorylation, disturbed the Bcl-2 family protein balance and activated caspase 12, caspase 9 and caspase 3. This could lead to apoptosis inhibition. Interestingly, it has been shown that Tau plays a beneficial role in preventing ER stress. Fourthly, Tau attenuated NF-κB activation and decreased pro-inflammatory cytokine (IL-6, IL-12, TNF-γ, and IFN-α) synthesis and reduced inflammation. Fifthly, in some cases (CP treatment), Tau metabolism and transport were affected, and overexpression of *TauT* in toxicant-treated animals showed a protective effect. This confirms that the protective action of Tau is concentration-dependent. In addition, the protective effect of Tau against damage caused by other toxicants, including heavy metals, cancer chemotherapy and other clinically relevant drugs, immunosuppressants, pesticides, insecticides and typical laboratory prooxidants, is associated with similar changes/restorations of various arms of the antioxidant defence network (Figure 1).

Finally, the protective effects of Tau against oxidative stress imposed by various toxicants were associated with the improvement of tissue integrity and organ functionality observed at the histological level, as well as at the level of specific markers of tissue damage (ALT, AST, ALP, GGT, etc., in plasma). Therefore, molecular mechanisms of the antioxidant protective actions of Tau are diverse, and direct radical scavenging, maintaining mitochondria integrity, membrane stabilisation, inhibition of ROS-producing enzymes and restoration of antioxidant enzymes activities in stress conditions, as described in this review, are of great importance. However, it seems likely that Tau’s interactions with the expression of transcription factors, including Nrf2 and NF-κB, and the improvement of adaptability to various stresses via vitagene activation deserve more attention.

## 8. Protective Effects of Tau in Stress Conditions of Poultry Production

Considering Tau as an important element of the antioxidant defence network, its protective effects under various stress conditions in poultry production have recently been reviewed [17], and can be summarised as follows. By decreasing oxidative stress in stressful conditions of poultry production, Tau was shown to help in fighting heat stress, immunological challenges or homeostasis disturbances associated with the increased stocking density of poultry [17]. Tau supplementation (2.50, 5.00, and 7.50 g/kg of the diet) was reported to improve the growth performance, antioxidant capacity, and lipid metabolism of commercial broiler birds [365]. Furthermore, dietary Tau supplementation was found to alleviate muscle loss in chronic heat stressed broilers via the prevention of oxidative stress and regulating the perk signalling [366]. Interestingly, it seems likely that Tau has a special protective role in the chicken gut, regulating mucosal barrier function and ameliorating LPS-induced duodenal inflammation in chickens [16].

The aforementioned data related to the protective actions of Tau in poultry under various stress conditions, medical applications [11,13,71] and research in other animals, including pigs [367,368,369,370], indicate that future Tau research in poultry should be conducted.

## 9. Conclusions

Tau is reported to improve the antioxidant defence networks in different ways.

- First, in some tissues (e.g., heart and eye), Tau can directly scavenge free radicals.
- Secondly, Tau participates in maintaining the integrity of electron-transport chain of mitochondria, the main sources of ROS in biological systems.
- Thirdly, Tau is shown to inhibit the activities of ROS-producing enzymes, including XO and NADP oxidase.
- Fourthly, by interacting with transcription factors (e.g., Nrf2 and NF-κB) and inducing AO enzymes, Tau can maintain the optimal redox status of the cell.
- Finally, by activating vitagenes, including SOD, HSP, thioredoxin, sirtuins, etc., Tau can provide additional protection in stress conditions and help in the creation of an effective adaptative response to stresses.

From the literature data analysed above, it could be concluded that:

- Tau plays a vital role in the AO defence network. However, the direct antioxidant effects of Tau in biological systems are limited.
- The stabilizing effects of Tau on mitochondria under various stress conditions, including commercial animal and poultry production, deserve more attention.
- A range of toxicological models clearly showed the protective antioxidant-related effects of Tau.
- Until now, major Tau applications have been related to human health, including the prevention/treatment of various diseases or decreasing the detrimental effects of various essential drugs used in human medicine.
- Furthermore, there are a range of publications confirming the protective effects of Tau (alone or in combination with other antioxidants) in various stress conditions of commercial meat and egg production.
- Anti-inflammatory and immunomodulatory properties of Tau are of great importance for veterinary medicine.
- Indirect antioxidant activities of Tau due to the modulation of transcription factors and vitagenes leading to the upregulation of the antioxidant defence network are likely to be major molecular mechanisms of Tau’s antioxidant and anti-inflammatory activities, and they deserve more attention and further investigations.

## Figures and Tables

**Table 1:** Regulatory roles of Tau in biological functions (adapted from [1,17]).

Function	Recent References
Antioxidant	[1,17,52,53,54]
Membrane stabilisation	[55,56,57]
Mitochondrial integrity maintenance	[11,13,58,59,60,61]
Vitagene activation	[1,62,63,64,65]
Bile acid conjugation	[66,67,68,69,70]
Ca homeostasis	[13,71,72,73]
FA metabolism/oxidation	[74,75,76]
Energy metabolism	[77,78,79]
Osmoregulation	[13,80,81]
Thermoregulation	[82,83,84]
Detoxification	[85,86,87]
Neuroprotection	[71,88,89,90,91]
Anti-inflammatory	[11,92,93,94,95]
Immunomodulation	[46,96,97,98,99]

## Abbreviations

AKT	protein kinase B
ALP	alkaline phosphatase
ALT	alanine aminotransferase
AST	aspartate aminotrasferase
AO	antioxidant
ALX	alloxan
BAD	BCL2 associated agonist of cell death
BCL2	B-cell lymphoma-2
CAT	catalase
COX2	cyclooxygenase-2
CP	cisplatin
CTSB	cathepsin B
CYP2E1	cytochrome P450 family 2 subfamily E member 1
8-OHdG	8 hydroxy 2 deoxyguanosine
DOX	doxorubicin
DPPH	1,1-diphenyl-2-picrylhydrazyl
EIF2α	eukaryotic initiation factor 2
ER	endoplasmic reticulum
ERK	extracellular signal-regulated kinase
ETC	electron-transport chain
FOXP3	Forkhead box protein P3
GGT	gamma-glutamyl transferase
GR	glutathione reductase
GRP78	78-kDa glucose-regulated protein
Grx	glutaredoxin
GSH	reduced glutathione
GPx	glutathione peroxidase
GST	glutathione *S*-transferase
HO-1	heme oxygenase 1
HSP	heat-shock protein
IκB	inhibitor of κB
iNOS	inducible nitric oxide synthases
IKK	IκB kinase
IFN-α	interferon alpha
IL	interleukin
JNK	c-Jun N-terminal kinase
Keap1	Kelch-like erythroid cell-derived protein with CNC homology (ECH)-associated protein 1
LDH	lactate dehydrogenase
LPO	lipid peroxidation
LPS	lipopolysaccharide
MAPK	mitogen-activated protein kinase
MDA	malondialdehyde
MTO1	a mitochondrial protein
NF-κB	nuclear factor-κB
NLR	NOD-like receptor
NLRP3	NLR family pyrin domain containing 3
Nox	NADPH oxidase
NQO1	NAD(P)H:quinone dehydrogenase 1
Nrf2	nuclear factor-erythroid-2 (NF-E2) and related factor 2
PAT1	DNA topoisomerase 2-associated protein
PI3-K	phosphoinositide 3-kinase
PPAR	peroxisome proliferator-activated receptor
PUFAs	polyunsaturated fatty acids
p53	tumour protein p53
RNS	reactive nitrogen species
RONS	reactive oxygen and nitrogen species
ROS	reactive oxygen species
SIL	silymarin
SOD	superoxide dismutase
STZ	streptozotocin
TAA	thioacetamide
Tau	taurine
TauCl	taurine chloramine
*TauT*	taurine transporter
TCA	tricarboxylic acid
TGF-β	transforming growth factor beta
TNF-α	tumour necrosis factor
TR	thioredoxin reductase
Trx	thioredoxin
UPR	unfolded protein response
XO	xanthine oxidase
